# The use of a surgical logbook to improve training and patient safety: A retrospective analysis of 6 years' experience in Bristol, UK

**DOI:** 10.1002/ski2.386

**Published:** 2024-06-06

**Authors:** Elizabeth Wasson, Charankumal Thandi, Adam Bray

**Affiliations:** ^1^ Department of Dermatology University Hospitals Bristol and Weston NHS Foundation Trust Bristol UK; ^2^ Department of Dermatology King's College Hospital NHS Foundation Trust London UK

## Abstract

**Background:**

Logbooks can be a useful educational tool. Although their use in medical training has been greatly explored, there is limited research into their application throughout a clinician's career. We created a surgical logbook to allow clinicians to record their skin surgery procedures and reflect on the histological diagnosis and clearance margins achieved.

**Objectives:**

We provide a retrospective analysis to evaluate the usefulness of the logbook as an analytical and educational tool in a dermatology department, particularly for maintaining exemplary complete excision rates.

**Methods:**

Data was collected from clinicians who conducted skin surgery in Bristol, UK. Cases were entered at the time of surgery, collecting data on body site, clinical margin, suspected diagnosis, type of procedure and closure. Following receipt of histological results, data on histological excision margins and tumour type was entered. Each clinician submitted data for a minimum of 3 months each year, and data collection ran from April 2012 until March 2018.

**Results:**

Data from 5161 excision surgeries was collected over the 6‐year period. On histological diagnosis, excisions constituted 50% Basal Cell Carcinoma (BCC), 12% Squamous Cell Carcinoma (SCC), 9% Malignant Melanoma (MM) and 1% Lentigo Maligna (LM), and 28% ‘Other’ (rarer malignancies, melanoma wide local excision scars and pre‐cancerous/benign lesions). The department was found to have good diagnostic concordance: BCC 92%, SCC 87%, MM 80% and LM 53%. Overall complete excision rate was high at 97.07%. The most successfully excised cancer was BCC (97.50%), then SCC (97.14%) MM (96.48%) and LM (95.23%). The department provided many complex surgeries including 8.3% flaps and 8.5% grafts. Average lesion diameter was 11 mm (range 2–90 mm). There was a significant improvement in excision rates seen over the years for BCC, MM and LM. Although there was no significant difference found for SCC, this group began the study with already high clearance rates.

**Conclusion:**

This surgical logbook supports improved training and continues professional development. We encourage more departments to use this logbook and share the data that they produce. This could improve their excision rates, patient experience, and save them up to £28 000 per year.

## Graphical abstract

We provide a retrospective analysis to evaluate the usefulness of the logbook as an analytical and educational tool in a dermatology department, particularly for maintaining exemplary complete excision rates in a dermatology department. We present how this logbook can be useful not only in dermatological training but also throughout a clinician's career. We also present the potential improvements in excision rates, patient experience and cost savings if this method is to be taken on by other departments.

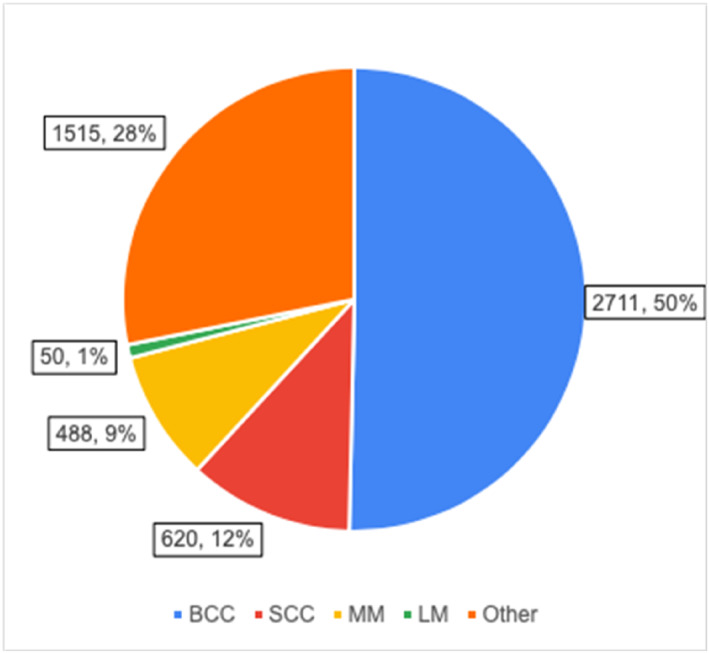

1

**What is already known about this topic?**

- Surgical logbooks are used throughout medical training to provide a record of adequate clinical exposure and reflection.
- However, logbooks usually record experience rather than outcomes, and this useful tool is not mandated during all clinical training or practice.
- The publishing of surgical data in national registries for some procedures has been shown to improve patient outcomes, but skin cancer surgery is not currently included.

**What does this study add?**

- We provide 10 years' experience and outcome data collected and analysed using a skin cancer surgery logbook and explain how this has improved complete excision rates and patient experience, and reduced healthcare costs.
- We demonstrate how the logbook can improve training and ongoing practice and detail a method of implementation that can be replicated in other departments.

## 2 INTRODUCTION

Surgical logbooks are used widely in medical training. They provide a way to record and reflect on procedures that have been undertaken, as well as ensuring adequate experience in the given speciality.^1^, ^2^, ^3^ Reflection and appropriate procedural exposure, whilst vital in training, are also important throughout a clinician's career. Monitoring the success of skin cancer excisions provides the opportunity to minimise secondary excisions, reduce unnecessary tissue removal, and facilitate optimal scars. Clear margin excisions also reduce pressure on public healthcare services by not exacerbating long waiting lists and capacity problems.

In 2012 an electronic surgical logbook was created in Bristol and made freely available via Internet download through the British Society for Dermatological Surgery.^4^ This logbook allows clinicians to keep track of their skin cancer excisions. Histological results can be entered, facilitating reflection on the diagnosis and clearance margins achieved. Results are automatically summarised for peer benchmarking and reflection on departmental outcomes, demonstrating that clinicians are meeting patient safety goals and the service is effective.

Many surgical logbooks described in the literature are focused on ensuring that trainees achieve the necessary experience for progression in their training.^2^, ^3^, ^5^ However, there is little literature on how effective the use of logbooks is beyond training and for recording patient relevant outcomes. This study was a retrospective audit and analysis to evaluate if this logbook can be used as an effective analytical and educational tool in a Dermatology department to try to achieve exemplary complete excision rates.

## 3 MATERIALS AND METHODS

### 3.1 Participants

All clinicians who conducted skin cancer surgery in Bristol were invited to use the surgical logbook, including medical and nursing staff.

### 3.2 Data collection

Clinicians logged their own consecutive excision procedures prospectively for suspected skin cancer at the time of surgery, from April 2012 until March 2018, giving information for 6 consecutive years of excisions. Each operator recorded cases for a minimum of three months each year, between 1^st^ April to 31^st^ March (but recording throughout the year was encouraged). Each year operators were chased several times until data was submitted. Delegated surgery was common, therefore surgeons using the logbook were instructed to enter the pre‐op diagnosis proposed by the clinician booking the operation.

Information on body site, clinical margin, and suspected diagnosis were logged. The type of procedure and closure were also recorded, giving an approximate measure of the complexity of the surgery. Once available, clinicians entered the histological results, including excision margins (deep and lateral) and tumour type. Trainees would enter their own data, and then discuss the data with their clinical supervisors.

### 3.3 Data analysis

Data collection and analysis was with Microsoft Excel using the bespoke Bristol Surgical Log v4.1 accessed via: https://bsds.org.uk/mohs‐surgery/national‐bsds‐logbooks/. This offers automatic data summarising and collation for various parameters of individual clinicians' practice.

Logbooks were kept individually on secure hospital servers then submitted for pooled departmental analysis and benchmarking. If there was missing data, logbooks were returned to operators for completion. Cases with incomplete results were excluded from analysis. A complete excision was defined as an excision with no involvement of the deep or lateral margin.

**FIGURE 1:**
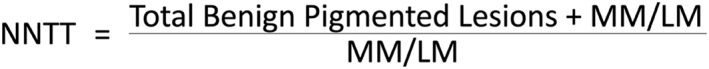
Equation used to calculate number needed to treat. MM, Malignant melanoma (invasive and in‐situ); LM, Lentigo Maligna.

The number needed to treat (NNT) can be calculated using benign and dysplastic naevi plus in‐situ and invasive melanoma as the numerator, and in‐situ plus invasive melanoma as the denominator (Figure 1). We used any pigmented lesions or forms of melanoma in the numerator (i.e. to include lesions such as seborrhoeic keratosis which may have been excised as possible MM) as this was more representative of clinical practice.^6^

## 4 RESULTS

### 4.1 Study population

**FIGURE 2:**
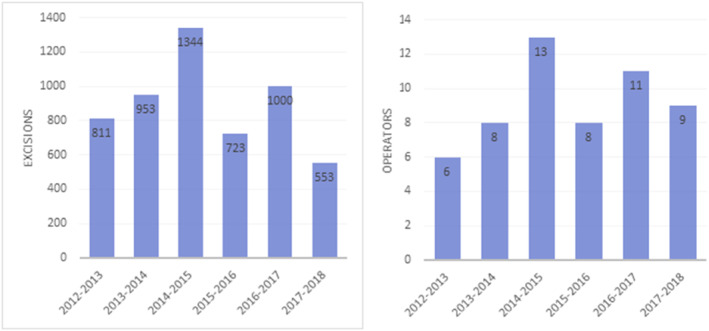
(a) Number of excisions entered into the log each year; (b) Number of operators that contributed data from the department each year of the log.

**FIGURE 3:**
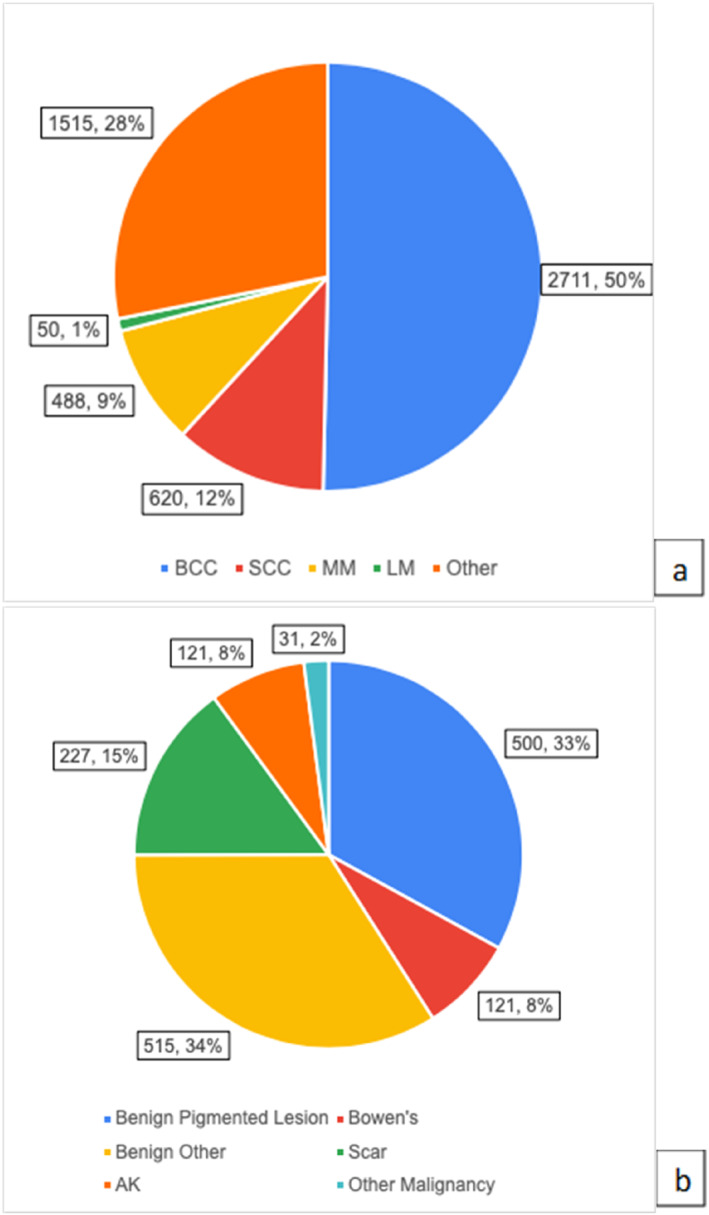
(a) A breakdown of histological tumour types excised; (b) A breakdown of histological diagnosis of excisions described as ‘Other’.

Contributors included Dermatologists, Dermatological Surgeons, Plastic Surgeons and Maxillofacial Surgeons, (Consultants and Speciality Trainees), and Clinical Nurse Specialists. This covered two Dermatology departments in neighbouring acute hospitals (University Hospitals Bristol NHS Foundation Trust, and North Bristol Trust). The number of clinicians that submitted data each year ranged from 6 to 13 with an average of 9 (Figure 2a). In total, 24 unique surgeons submitted data, comprising the vast majority of those undertaking skin surgery for the dermatology service. The number of trainees, nurses, and other specialists employed and contributing varied each year as personnel changed. Contracted Plastic Surgeons working within the Dermatology department (approximately 5 individuals per year) did not usually contribute their own figures, therefore in 2014–2015 a validation exercise was completed by collecting all the cases on their behalf. We did not find any significant variation from the other years' outcomes. Five thousand one hundred and sixty‐one excisions were entered between 2012 and 2018, averaging 860 per year (Figure 2b). Three thousand eight hundred sixty nine were skin cancers (Figure 3a).

### 4.2 Tumour type

The most common histological diagnosis was basal cell carcinoma (BCC) at 50.3%, with squamous cell carcinoma (SCC) 11.5%, MM 9.1% and lentigo maligna (LM) 0.9% (Figure 3a). The 28.1% remaining constituted rarer malignancies, melanoma wide local excision scars, and pre‐cancerous or benign lesions (e.g. moles) excised to exclude skin cancer (Figure 3b).

**FIGURE 4:**
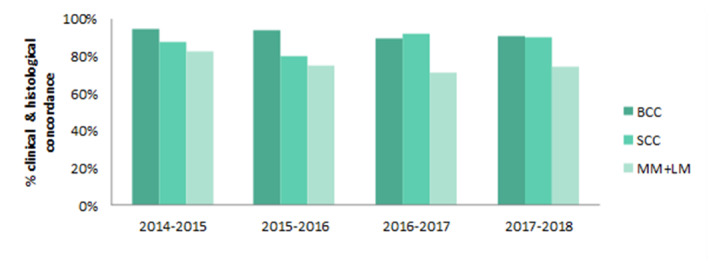
Clinical and histological diagnostic concordance for each diagnostic group through the study period (e.g. clinical diagnosis Malignant Melanoma (MM) + Histological seborrhoeic keratosis = non = concordance).

Most skin cancers underwent excisional rather than incisional biopsy and data showed that clinicians were good at predicting lesions that would be malignant. From 2014 onwards we compared how many cancerous lesions found on histology had been correctly diagnosed prior to excision. BCC was found to be the most successfully predicted (92.6%) closely followed by SCC at 87.2% diagnostic concordance (Figure 4). Comparing these figures to 2014 and 2016 British Society of Dermatology Audits, our diagnostic concordance was slightly lower than averages for BCCs (94%–96%) and significantly higher than those found for SCCs (67%–80%).^7^, ^8^ MM and LM were found to have a diagnostic concordance of 80.0% and 53.2% respectively. When comparing suspected with histologically diagnosed MM/LM (invasive or in‐situ), the NNT was 2.53.

### 4.3 Excision data

**FIGURE 5:**
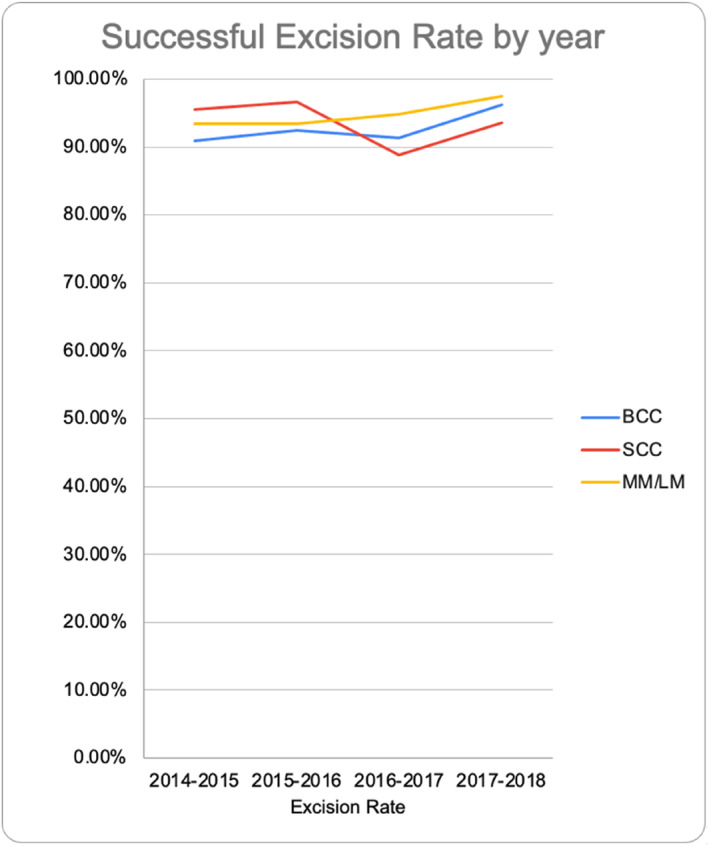
Successful skin cancer complete excision rate (at both lateral and deepp margins) rates over time, split by malignancy type.

The complete excision rates remained high throughout the use of the logbook, averaging 97.1%, with 0.1% of cases showing involvement at both lateral and deep margins. Individual surgeon excision rates ranged from 79.5% to 98.7%. The most successfully excised cancer was BCC, with a complete excision rate of 97.5%. This was closely followed by SCC at 97.1%, MM at 96.5% and LM at 95.2%. Figure 5 demonstrates the high excision rates achieved throughout the study period.

Re‐excisions were infrequent, making up just 5.6% of procedures undertaken by the department with 89.2% being primary excisions (wide local excisions for melanoma were recorded separately from this category). This suggests that the department's high complete excision rates correlate well with actual cure rates. The most common lesions to be re‐excised were BCCs (58.4%) followed by SCCs (31.0%) and MM (3.6%).

**FIGURE 6:**
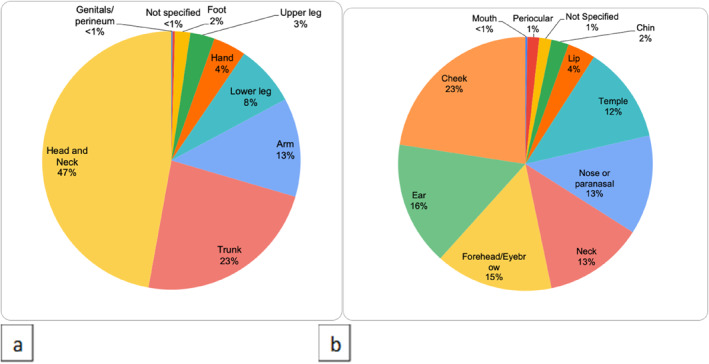
(a) Breakdown of anatomical body site of excisions performed; (b) Further Breakdown of anatomical site of head and neck lesions excised.

Lesions ranged from 1 to 90 mm in diameter, with the average size being 11 mm. Procedures comprised 8.3% local flaps and 8.5% skin grafts, and 47% were from the head and neck (Figure 6a).

## 5 DISCUSSION

The method of logging cases was easily accessible with reliable technology, thus reducing barriers to participation and maximising engagement. This has produced a large and robust dataset, demonstrating repeatability and longevity. We propose that data quality is high, due to the operating surgeons completing the data entry, and being motivated to complete the logbook for their own development and peer review.

The complete excision rates in our study were high. This could not be completely explained by a skewed casemix towards simpler cases. There was a higher proportion of more complex cases than seen in other papers by Dermatologists (17% local flaps or skin grafts),^7^, ^8^ but lower than some Plastic Surgery centres (Table 1).^9^ A systematic review by Nolan et al found that Dermatologists performed 9.0% complex procedures (flaps or grafts) and Plastic Surgeons 39%. This study also found 93.8% complete excision rates for Dermatologists and 90.6% complete excision rates for Plastic Surgeons.^10^ Whilst the majority of our excisions were recorded by Dermatologists, we also had some Plastic and Maxillofacial Surgeons that submitted to our database. In our study individual surgeons did not need to share their figures publicly, except with colleagues in their own department. The aim of the logbook was for surgeons to reflect on their own work and compare to departmental/national averages rather than to publish individual results in the public domain. However, the habit and system could potentially be used as the foundation for a national registry in future. We observed that individuals with lower performance tended to take action themselves to make improvements for example, increasing clinical excision margins or reducing the complexity of the cases they took on.

**TABLE 1:** A literature search of other published audits of melanoma and non‐melanoma skin cancer excision rates.^7^, ^8^, ^9^, ^22^, ^23^, ^24^, ^25^, ^26^, ^27^

	First author	Year	Country	Setting	Tumour types	% complete excision rates
1	E Delaney	2012	United Kingdom	Primary and secondary care	SCC	87.5%
2	T Brian	2018	New Zealand	Primary and secondary care	Melanoma	90.4%
3	H Haque Hussain	2009	United Kingdom	Secondary care	SCC	91.5%
4	V Malik	2010	Ireland	Secondary care	BCC	92.6%
5	G Nolan	2022	United Kingdom	Secondary care	BCC and SCC	94.7% (BCC)
						92.3% (SCC)
6	A E Macbeth	2009	United Kingdom	Secondary care	BCC	94.0%
7	D Wen	2020	New Zealand	Primary care	BCC and SCC	96.6%
8	D J Keith	2020	United Kingdom	Secondary care	BCC and SCC	97.0%
9	D J Keith	2017	United Kingdom	Secondary care	BCC and SCC	97.7%

### 5.1 Training

The use of a surgical logbook has become standard practice within the department. It is used for self‐reflection and review at an annual appraisal for both senior surgeons and trainees. There is now an expectation that trainees include their anonymised surgical logbook as part of their electronic training portfolio. The logbook is also used by many other departments within the United Kingdom.

Logbooks are clearly useful in medical training. They provide a record of adequate exposure to an area of practice and act as an important reflective tool. Logbooks can also be important to demonstrate that a centre is providing adequate supervision and opportunities for trainees.^3^ Using this logbook, results were fed back to each surgeon annually with the department average and peer figures which led to improvement or change of practice. This not only allows clinicians to review the ‘success’ of their excisions, but also provides a record of surgeries they have performed. Furthermore, reflection on any differences between the suspected pre‐operative diagnosis compared to the confirmed histological diagnosis was found to be a useful tool particularly for trainees, but also for experienced surgeons (e.g., Bowen's disease or BCC clinically manifesting as SCC).^5^

Farrant et al surveyed Dermatology registrars, asking what attributes they looked for in their teachers, with the highest rated concerning providing feedback and answering questions. This logbook provides an avenue for trainees to get feedback on their performance that can be used in case‐based discussions and assessments. 80% of respondents felt it was important that their trainers audit their own practice, while 90% believed it was important that they discussed their own limitations.^11^ The importance of trainers reflecting and being a good role model has been seen in many studies.^12^, ^13^ This logbook therefore provides the opportunity for consultants to audit their work and thus become better teachers.^14^ Furthermore, by providing individual surgeons with accurate complete excision data they can share this with patients to inform decision making.

Collation of these logbooks provides departments with quality data on Dermatological surgery activity. This can highlight topics for targeting staff education (such as reducing excisions of benign pigmented lesions), or areas for which greater funding is required (such as more complex surgeries). This information has been used locally to demonstrate a high‐quality cost‐effective service to those funding care, including compliance with avoiding non‐funded procedures on benign lesions.

### 5.2 Patient outcomes registries and future developments

There are limited publications on the use of logbooks to improve surgical patient outcomes. However, medical registries are similar, providing data on the effectiveness of healthcare in a population. The National Surgical Quality Improvement Programme across North America has demonstrated how hospitals have saved up to $4.5million a year in reduced post operative complications.^15^ This demonstrates the clear benefits of departments publishing their data.

The UK National Joint Registry collects large amounts of surgical data, which was novel at its inception in 2000. Individual outcomes for each surgeon as well as national benchmarking data led to publishing significant findings such as the huge differences in revision rates from different joint materials, and the benefits of patient reported outcome measures when looking at post‐op analgesia.^16^ Although many originally thought it would be too time consuming and costly, over time the reduced revision costs have far outweighed the costs of input. Furthermore, information technology advances should make data collection much easier and potentially more automated, and outcomes more useable as part of appraisals and revalidation.

Currently, there are no standardised outcome sets for skin cancer surgery in the UK. However there are new guidelines being developed for use in trials and clinical practice.^17^ We hope that publishing our data will provide further insight, and there is potential to utilise the logbook to create a larger scale registry in future.

### 5.3 Benchmarking

Complete excision rates may be adversely affected by clinicians who perform less surgery, as well as clinicians who specialise in more complex procedures. Operators carrying out more complex procedures might be expected to see more complex tumours with a higher likelihood of incomplete excision. Therefore, the case mix and scope of practice must be included with any outcome figures, so that meaningful comparisons can be made, and operators with similar casemix can still benchmark against each other.

### 5.4 Impact of COVID‐19

Since the study period Covid‐19 reduced theatre time and training opportunities. Reduced face‐to‐face clinic contact led to more teledermatology: patients emailed lesion photographs, which were uploaded to hospital records. Although some patients required further review and dermoscopy, many patients were able to have surgery directly booked. The logbooks have therefore been even more valuable, as clinicians are now able to look back at photographs after the procedure. This provides an additional learning opportunity to compare how their initial clinical diagnosis matched histopathological diagnosis. Subsequently pre‐operative images have been mandated for all cases to reduce risk of wrong site surgery, further enhancing the educational benefits.

### 5.5 Patient impact

Treatment options for incompletely excised tumours have an impact on patient experience. Since the treatment has failed, additional clinical and patient time will usually be needed to explain further treatment options so the patient can make an informed choice of how to proceed. Options are usually: further surgery (at times Mohs Micrographic Surgery [MMS]), radiotherapy, additional clinic visits for monitoring, or no additional treatment/monitoring. The latter brings an accepted increased risk of recurrence that may eventually lead to any of the above options needing to be reconsidered. These additional discussions and decisions or lack of closure to the episode can cause worry to patients and caregivers. This impact is hard to quantify but seems desirable to minimise.

### 5.6 Economic impact

The effect of incomplete excisions on out‐patient clinic and administrative clinician time is hard to quantify. However, we can estimate the impact on theatre time and procedure costs.

Published evidence of complete excision rate audits show a range of 87.5%–97.7% (Table 1). Our results were towards the higher end of this range. If we assume that all incompletely excised tumours undergo subsequent standard re‐excision we can make a rough estimate of excess costs. In reality, a proportion would have higher cost treatments such as radiotherapy, or MMS, or lower cost options such as clinic monitoring.

If we consider a fictional department with similar procedure numbers/case mix to ours, but performing at the lower end of the range, the difference in extra re‐excision procedures (i.e. between an 87.5% and a 97.1% complete excision rate) could amount to up to approximately 350 incomplete excisions over our study's duration. Assuming that an average excision takes about 45 min and a complex procedure 60–90 min, we can use 50 min as the average according to the casemix we saw in our cohort. Therefore, roughly 300 h of theatre time could be saved over the course of this 6‐year study, by increasing the complete excision rate to our figure. Given estimates suggest that UK theatre time may cost £561 per hour, this could equate to over £28 000 in savings for just one department each year.^18^ We appreciate that many factors influence the excision rates of a department. However we postulate that regular study and discussion of this topic creates an expectation of a high complete excision rate and a culture of self‐improvement, thereby maintaining high standards.

### 5.7 Limitations

Our NNT of 2.53 was lower than expected from the literature, and a previously published figure of 3.19 from our unit. This could reflect more certainty of a melanoma diagnosis arising from late presentation. Previously we found a mean Breslow thickness of 1.29 mm for invasive melanoma and an invasive: in‐situ melanoma ratio of 1.6 for excisions in our service, which does not support that hypothesis, but rather suggests relatively early diagnosis.^8^ The increasing rates of skin cancer in our region could have had an impact on increasing pre‐test probability. Also dermoscopy use and training increased over this period, which may have excluded more benign lesions prior to excision.

There was a risk of selection bias from self‐reporting, however this was reduced by asking clinicians to submit at least 3 months of consecutive data, and to enter cases prospectively at the time of surgery before outcomes are known.

Pre‐operative suspected diagnosis could potentially be edited following histology. However, in our team‐delegated system, surgeons were told to record the suspected diagnosis of whoever requested the surgery, often not themselves, so diagnostic accuracy data would only reflect summary departmental practice. There would be little incentive for surgeons to alter that data.

Although any surgeon might potentially feel anxious about their outcomes being scrutinised, no specific sanction for poor results has been suggested or required for any surgeon in this study. Feedback meetings were always carried out in a supportive and educational fashion, with the aim of minimising anxiety, and maximising engagement and learning. Also, incomplete excisions of skin cancer are all flagged automatically from the histopathology results and discussed at a weekly skin cancer multidisciplinary meeting. Therefore, significant outlier surgeons would become obvious in any case.

Completing the logbook required extra time. Clinicians had to input their own data and look up their own histology. However, this was felt to be worthwhile as it gave clinicians the opportunity to see their data and reflect (and review case notes or photographs if needed). As software interoperability advances, logbooks could become more accessible and convenient for clinicians and reduce manual data entry. Ideally this should be integrated with routine data collection in hospitals. There are also now several applications developed for trainees to record their surgical experience easily.^19^

## 6 CONCLUSION

The surgical logbook provides a rich learning resource. It can positively impact training and ongoing practice. If used nationally, especially as a regular tool, it could improve complete excision rates, patient experience, and reduce healthcare costs. Publication of results and trainee data could lead to a virtuous circle of healthy competition between departments leading to improved training opportunities, higher complete excision rates, and a reduction in unnecessary procedures.^20^, ^21^ We would encourage more departments to use this logbook and share information that they discover.

## CONFLICT OF INTEREST STATEMENT

None to declare.

## AUTHOR CONTRIBUTIONS

**Elizabeth Wasson**: Data curation (supporting); formal analysis (lead); investigation (lead); methodology (lead); project administration (lead); writing – original draft (lead). **Charankumal Thandi**: Supervision (supporting); writing – review & editing (supporting). **Adam Bray**: Conceptualization (lead); data curation (lead); supervision (lead); writing – review & editing (lead).

## ETHICS STATEMENT

Not applicable.

## Data Availability

The data underlying this article will be shared on reasonable request to the corresponding author.
